# Retraction: Aqueous room temperature mono-dehydration of sugar alcohols using functionalized yttrium oxide nanocatalysts

**DOI:** 10.3389/fchem.2024.1481134

**Published:** 2024-08-22

**Authors:** Frontiers Editorial Office

The Journal retracts the 2020 article cited above.

Following publication, concerns were raised about an unacceptable level of similarity between the article cited above in Frontiers in Chemistry and the Green Chemistry publication: Green Chem., 2020, 22, 5333–5344, by the same authors.

An investigation was conducted in accordance with Frontiers’ policies, whereby it was confirmed that the Green Chemistry’s article was published while the Frontiers in Chemistry submission was still in review.

Following the Committee on Publication Ethics guidelines, this article, published most recently is retracted. This retraction was approved by the Chief Editors of Frontiers in Chemistry and the Chief Executive Editor of Frontiers. The authors did not respond to our communication regarding the retraction.

